# Time Spent in a Maternity Pen during Winter Influences Cow and Calf Behavior in Pasture-Based Dairy Systems

**DOI:** 10.3390/ani12121506

**Published:** 2022-06-09

**Authors:** Fabiola Matamala, Helen Martínez, Claudio Henríquez, Pilar Sepúlveda-Varas

**Affiliations:** 1 Escuela de Graduados, Facultad de Ciencias Veterinarias, Universidad Austral de Chile, Valdivia 5090000, Chile; fabiolamatamalahernandez@gmail.com; 2 Escuela de Graduados, Facultad de Ciencias Agrarias y Alimentarias, Universidad Austral de Chile, Valdivia 5090000, Chile; helenmichellemartinez@gmail.com; 3 Instituto de Farmacología y Morfofisiología, Facultad de Ciencias Veterinarias, Universidad Austral de Chile, Valdivia 5090000, Chile; claudio.henriquez@uach.cl; 4 Instituto de Ciencias Clínicas Veterinarias, Facultad de Ciencias Veterinarias, Universidad Austral de Chile, Valdivia 5090000, Chile

**Keywords:** calving management, welfare, cattle, pasture-based systems, winter

## Abstract

**Simple Summary:**

One of the major challenges of spring calving pasture-based systems in temperate regions is the exposure of periparturient dairy cows and their newborn calves to cold and wet winter conditions. We investigated whether moving precalving cows from an outdoor paddock to an indoor maternity pen affects the behavior of the cow and her newborn during winter and if this was influenced by the timing relative to calving. Our results indicated that cows housed in a maternity pen spent more time lying and ruminating compared with cows kept in an outdoor paddock. Moreover, newborn calf vitality improved when cows were moved to a maternity pen 3 weeks before calving when compared with those moved during the week before calving or those that remained in an outdoor paddock precalving. This information may aid in the design of calving management systems that can minimize any negative effects of inclement winter weather on cow and calf welfare in seasonal, year-round pasture-based systems.

**Abstract:**

Our study compared the behavior of prepartum dairy cows that either remained in an outdoor paddock until calving (OP) during winter or were moved to an indoor maternity pen either early (EM) or late (LM) relative to calving. Forty-two multiparous Holstein cows were divided into three treatments (OP, EM, or LM) and monitored from 3 weeks before to 1.5 h after calving. Cows in EM and LM were moved to a maternity pen starting at week three and week one before the expected calving date, respectively. We assessed the cleanliness of the cows at calving, immunoglobulin G concentration in colostrum, and the behavior and vitality of calves across treatments. Cows spent more time lying in EM compared to OP and LM during the weeks −3 and −2 relative to calving, but lying time was increased in LM cows compared with OP cows during the week −1 relative to calving. Prepartum rumination time was lowest in OP cows but not different between EM or LM. Calves from OP cows spent more time lying and had lower vitality after calving than those from LM and EM cows, respectively; calves from EM and LM cows were intermediate for lying and vitality, respectively, but did not differ from either group. The cleanliness was greatest in cows that calved indoors (EM or LM); nevertheless, precalving management did not affect the IgG concentration in colostrum. Our study demonstrates that, in comparison with OP, EM and LM have positive implications for the welfare of the dam and its newborn calf during winter.

## 1. Introduction

The time around calving has been recognized as a critical period for the life and welfare of the dam [1,2] and its newborn calf [3,4]. In pasture-based systems located in temperate regions, such as southern Chile or New Zealand, parturition management aims to ensure that most cows calve during the spring calving season (from late winter to early spring) to allow for maximum pasture utilization to support lactation and thus minimize feeding costs [5]. In this type of dairy production system, during the prepartum period (i.e., three weeks before the expected calving date), cows are commonly located in outdoor paddocks with or without access to pasture, where they remain until calving [6,7]. Because the calving season typically coincides with cold and wet weather conditions [8], it has been recommended that outdoor calving paddocks should provide dry ground, shelter, and protection to dairy cattle during periods of winter weather [9]. However, despite these recommendations, shelter (natural or artificial) is rarely provided in practice [10]. For instance, a survey restricted to pasture-based dairy farms in southern Chile found that about half of the farms did not have indoor maternity areas and left the prepartum cows in small “sacrifice” outdoor paddocks without shelter during winter months [7]. 

The accumulated body of evidence indicates that clean, dry, well-bedded, and comfortable resting surfaces are important to dairy cows, particularly during the prepartum and calving periods, as they increase lying time, ensure hygiene, facilitate the calving process, and decrease the risk of illness after calving for both the cow and calf [11]. Thus, the use of maternity facilities in pasture-based systems could be an attractive option for farmers because it provides a clean and dry environment that minimizes stress and ensures the comfort of the cow and its newborn calf [12,13]. Moreover, it could promote better calving supervision to ensure assistance at calving if needed [14,15]. This could be particularly important because problems related to parturition may increase the risk of stillbirth, low calf vitality, and lower passive transfer of immunity [16,17]. However, the use of calving facilities has received less research in pasture-based systems than in confinement systems.

It has been recommended that cows should be moved to a calving facility within 1 or 2 days before calving to allow animals to adapt to their new environment [14,18]. Because environmental winter factors in temperate areas, such as rain and low temperatures, can reduce lying time [8,19,20,21], it may be beneficial to move prepartum cows from an outdoor paddock to an indoor calving area before calving to ensure the protection from adverse weather. A previous study has shown that when managed in outdoor paddocks without access to shelter during winter, cows spend less time lying, body cleanliness is impacted, and their blood NEFA concentrations increase prepartum [22]; these results are of interest provided that decreased lying times in prepartum dairy cows is associated with lower calf survival at parturition [23]. Moreover, elevated prepartum serum NEFA concentrations are associated with dystocia [24] and stillborn calf [23]. To our knowledge, there have been no studies directly evaluating the timing of access to a maternity pen on the behavior of dairy cows and their offspring exposed to winter weather in a temperate climate.

This study has two main objectives: (1) to investigate differences in lying, rumination, and activity behavior of prepartum dairy cows that remain in an outdoor paddock until calving (OP) or were moved indoors to a maternity pen either 3 (EM) or 1 week (LM) before calving during winter, and (2) to assess the effect of these different treatments on both dam and calf behavior immediately after calving. In addition, we assessed the cleanliness of the cows at calving and IgG concentration in colostrum. 

## 2. Materials and Methods

### 2.1. Animals, Management, and Experimental Design 

The study was carried out at the Austral Agricultural Experimental Station of the Universidad Austral de Chile in Valdivia, Chile (39°47′46″ S, 73°13′13″ W) between June to September 2019 (Southern Hemisphere winter). Valdivia has a Temperate Oceanic Climate (Cfb: temperate rainy climate in winter) according to Köppen–Geiger classification, as described by Sarricolea et al. [25].

A total of 42 clinically healthy multiparous late pregnant Holstein cows (mean ± SD parity = 3.1 ± 1.6; BW = 624 ± 72 kg; BCS = 2.7 ± 0.3 (5—point scale, [26]) were used. Cows were selected from a group of dry cows based on expected calving dates. Cows were managed according to the standard operating procedures for this facility, which included a typical herd of grazing cows under a seasonal spring calving system. Cows were dried-off approximately 8 weeks before their expected calving date. At the beginning of the dry-off period, as a preventive measure for lameness, cow’s feet were trimmed by a trained veterinarian. From the beginning of the dry period until approximately three weeks before the expected calving date (far-off period), cows were maintained in outdoor paddocks with pasture (mixture of grasses and legumes) and fresh water ad libitum. The stocking rate in these outdoor paddocks was maintained at approximately 20 to 25 cows/ha, but this was dynamic as cows came and left the outdoor paddock depending on their expected calving date. One week before enrolment, a trained veterinarian performed a general clinical examination of all cows. Cows clinically healthy pre-, during, and post-calving were used in this study, whereby no clinical signs indicative of illness or lameness were observed, and no cows required assisted calving. The cows selected for the study were marked with numbers using hair dye (White Bleach and Oxi-Cream, Elgon, Italy). After calving, cows remained with their newborn calf for at least 1.5 h (range: 1.5 to 12 h). Then, the calf was moved to the calf barn by the farm personnel, and the cow was integrated into the lactating group.

Cows were enrolled in the study three weeks before their expected calving date and allocated to 1 of 3 treatments based on the expected calving date. Provided the limited availability of maternity pens, the sample size was restricted to a total of 14 cows per treatment. 

**Figure 1:**
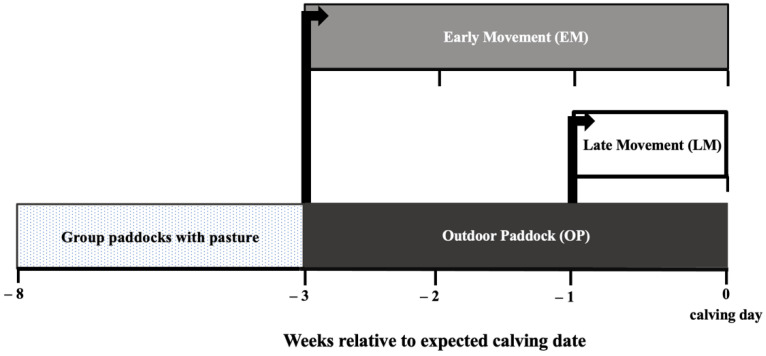
Diagram of the study design. During the far-off period, cows were grouped together in an outdoor paddock with pasture. Three weeks before the expected calving date, cows were individually allocated to 1 of 3 treatments: Outdoor paddock (OP; black bar), where cows were individually kept in an outdoor paddock from 3 weeks before until the day of calving; Early movement (EM; grey bar), where cows were individually housed in a maternity pen from 3 weeks before until the day of calving; and, Late movement (LM; white bar), where cows were individually kept in an outdoor paddock during weeks −3 and −2 and moved to individual indoor maternity pens during week −1 relative to calving until the day of calving.

The treatments were as follows (Figure 1):

1. Outdoor paddock (OP): Cows were individually kept in an outdoor paddock for the last three weeks before their expected calving date until calving day and exposed to natural winter weather conditions (i.e., rain, wind, and cold).
2. Early Movement (EM): Cows were individually housed in a maternity pen for the last three weeks before their expected calving date until calving day.
3. Late Movement (LM): Cows were individually kept in an outdoor paddock during weeks −3 and −2 and moved to individual indoor maternity pens during the week −1 relative to calving until the day of calving.

Each outdoor paddock measured 3.5 × 5 m and had a bare soil surface with no grass cover, reduced water infiltration, and moderate mud content. Outdoor paddock set-up allowed for visual, auditory, olfactory, and limited tactile contact between cows because an electric fence separated the experimental outdoor paddocks. The amount of mud was controlled through a boot test used previously by our research group [22] and adapted from Chen et al. [27]. To do this, the researcher stands on 2 spots inside of each outdoor paddock, and the marks left on the boots indicate how far mud comes up. The boot test consists of a three-point scale: 1 = dry soil (mud does not cover the boots); 2 = muddy soil (boots covered with mud below ankle level); and 3 = very muddy soil (mud-covered boots above ankle level).

Each indoor maternity pen used measured 3.5 × 5 m and consisted of an aluminum roof, wood walls, and a thick layer of sawdust (10 cm deep approximately) on concrete flooring. In this area, manure and urine were raked and removed daily after the morning feeding. Sawdust was changed or added daily or when necessary to ensure that each lying space was clean and dry.

Cows were individually fed twice daily at approximately 09:00 and 15:00 h; the rations were provided in a feed bin placed in the corner of the outdoor paddock or maternity pen. The ration was formulated following NRC [28] guidelines and consisted of approximately 26 kg of grass silage per day on an as-fed basis (27% DM, 14% CP, 49% NDF, and 2.4 Mcal/kg; reported on a DM basis) and approximately 3 kg of commercial concentrates per day on an as-fed basis (87% DM, 22.5% CP, 12.0% NDF, and 11.56 Mcal/kg; reported on a DM basis) with anionic mineral mix (Mg 4.0%, Cl 33.0%, S 2.6%, Ca 0.7%, K 0.3%, 2 mg/kg Na, 1050 mg/kg Cu, 2100 mg/kg Mn, 3500 mg/kg Zn, 140 mg/kg I, 13 mg/kg Co, 10 mg/kg Se). Clean, fresh water was provided *ad libitum* in a water trough (600 L water trough).

### 2.2. Weather Conditions Measurements

The daily measurements of rainfall (mm), air temperature (°C), relative humidity (%), and wind speed (m/s) were obtained from a weather station (A720, ADCON Telemetry GMBH, Klosterneuburg, Austria) located 1 km from the research location. The daily measurements of ambient temperature and relative humidity inside the maternity pen were recorded using electronic data loggers (RC-4HC, Elitech Technology, Inc., Milpitas, San Jose, CA, USA). All measures of weather conditions were included to describe the weather conditions in this study.

### 2.3. Behavioral Measurements

Prepartum lying behavior was recorded using electronic data loggers (HOBO Pendant G Acceleration Data Logger; Onset Computer Corp, Bourne, MA, USA) attached to the hind leg of each cow using a flexible bandage. The loggers were removed weekly from the animals for data download and then reattached to the leg. The data logger was set to record the y-axis at 1 min intervals for consecutive hours, and lying data were processed using the cutoff point validated by Ledgerwood et al. [29]. This information was used to determine whether the cow was standing or lying and subsequently was used to calculate daily lying time, number of lying bouts (i.e., frequency of transitions from lying to standing positions), and duration of lying bouts (minutes lying per day/the number of lying bouts per day). Lying down events shorter than two minutes were removed from the data following the recommendation of Mattachini et al. [30].

Prepartum rumination and neck activity behavior were measured using the Hr-Tag collars (SCR Engineers Ltd., Netanya, Israel) as described and validated by Schirmann et al. [31]. These collars consisted of a microphone to monitor rumination time and an accelerometer that quantifies neck activity. Rumination time was recorded in minutes per 2 h interval, and neck activity data were also recorded every 2 h as an arbitrary number. Data were transferred and stored in the control unit via radio frequency and downloaded daily to the database. This information was used to determine total rumination time and neck activity per cow per day.

Calving time was defined as the time when the calf was fully expelled from the cow [32]. The individual behavior of the cows and their calves during the first 1.5 h after calving were video recorded. Outdoor paddocks and maternity pens were equipped with one camera each (Ezviz; Model CS-CV310-A0-1B2WFR, City of Industry, CA, USA) that were mounted on wooden poles 2.2 m above the ground to visualize the complete individual area in the outdoor paddock or pen. The area under observation was naturally lit during daylight hours (08:00 to 17:59 h), and infrared lighting was used for night-time recording (18:00 to 07:59 h). The signal from the cameras went through an NVR recorder (Ezviz; Model Wi-Fi EZVIZ X5C). The behaviors posture, maternal behavior, comfort-related behavior, and calf vitality are described in Table 1 were continuously monitored at one-second intervals by one trained observer using the Behavioral Observation Research Interactive Software (BORIS, http://www.boris.unito.it/; access date: October 2019 [33]). Inter-observer agreement was calculated through the intraclass correlation coefficient (ICC = 0.96).

### 2.4. Cow Cleanliness and Immunoglobulin G Colostrum Concentration

Cow cleanliness was evaluated by a single observer at enrollment in the study (3 weeks before expected calving date) and immediately after calving. Cleanliness scores of the tailhead, upper leg (thigh), ventral abdomen, udder, and lower hind leg were evaluated using a 5—point scale, where 1 = clean to 5 = dirty [35].

Colostrum samples of 50 mL were taken from each cow at first milking (between 3 to 24 h after calving) and immediately frozen at −20 °C until analysis. The IgG colostrum concentration was measured using a commercially available bovine IgG ELISA kit according to the manufacturer’s instructions (ab205078, Abcam, Cambridge, UK). In brief, after thawing at room temperature, colostrum samples were serially diluted in ELISA wash buffer to final dilutions of 1:1,000,000.

### 2.5. Data Handling and Statistical Analyses

Statistical analyses were performed through R (version 4.0.3; https://www.r-project.org/; access date: October 2020) with linear mixed-effects models using R packages lme4 [36] and using the cow as the experimental unit. Models were checked to ensure normality of residuals, and the appropriate covariance structures were selected based on the lowest Akaike information criterion (AIC). Significant effects were defined as *p* < 0.05, *p* < 0.001 and tendencies were considered *p* < 0.10.

Descriptive statistical analyses (median and range) were performed to summarize the weather conditions. Three cows were excluded from the analysis of prepartum behavior, cleanliness score, and IgG colostrum concentrations because they calved before their expected calving date (missing more than 1 week), and thus, they did not complete the 3 weeks prepartum period in their respective treatment (OP, *n* = 1; EM, *n* = 1; LM, *n* = 1). Therefore, the final analysis of prepartum behavior, cleanliness score, and immunoglobulin G concentration in colostrum included 39 cows (13 cows per treatment).

In order to evaluate prepartum behavior, the lying, rumination, and activity behavior data were summarized into 3 periods based on week relative to calving: week −3 (days −21 to −15), week −2 (days −14 to −8), and week −1 (days −7 to −2). The data were analyzed using linear mixed-effects models, considering the treatment (OP, EM, and LM), and period (week −3, week −2, and week −1) as fixed effects and the cow as random effect. To evaluate cow cleanliness, the model considered the treatment (OP, EM, and LM), and observation period (at enrollment and after calving) as fixed effects, while the cow was considered as random effect. A linear mixed effects model was also used for evaluating IgG concentration in colostrum (mg/mL), considering the treatment (OP, EM, and LM) as fixed effect, whereas the calving time (day: from 08:00 to 17:59 h; night: from 18:00 to 07:59 h), and colostrum sampling time (>12 h and <12 h) were considered as random effects.

An additional 10 cows (and their newborn calves) were excluded from the analysis of behavior after calving due to technical problems with the video recordings (OP, *n* = 4; EM, *n* = 3; LM, *n* = 1) or having a stillborn calf (EM, *n* = 2). Hence, 29 cows and their calves were included (OP, *n* = 9; EM, *n* = 8; LM, *n* = 12). Because all calves performed a standing attempt, this behavior was calculated as the time from birth to the onset of this behavior within the first 1.5 h after calving (latency). However, because other vitality behaviors (e.g., successful standing, suckle attempt, or successful suckling) were not performed by all calves, we decided to analyze them as behavior performed (yes/no).

To examine the effect of treatment (OP, EM, and LM) on both dam (latency to stand after calving, latency to groom her calf, and time spent grooming her calf) and calf behaviors (lying time, frequency lying bouts, and latency to stand attempt) linear mixed-effects models were used. Characteristics of the calving (parity of the cow, cow body condition score at calving, and sex of the calf) were considered random effects.

We analyzed the association between treatment (OP, EM, and LM) and performed behaviors (dam: lying after calving and self-grooming; calf: successful standing, suckle attempt, and successful suckling) using a Fisher’s exact test R package RVAideMemoire [34].

## 3. Results

### 3.1. Weather Conditions

During the study period, daily median precipitation was 2.4 mm (range: 0 to 62.8 mm), the daily temperature reached 7.6 (range: 3.4 to 12.8 °C), and daily relative humidity was 83.4% (range: 60.8 to 96.9%), and daily wind speed was 2.9 m/s (range: 0 to 17.6 m/s). Inside the maternity pen, daily temperature and relative humidity averaged 9.5 °C (range: 4.2 to 13.3 °C) and 85.6 ± 0.7% (range: 61.3 to 92.4%), respectively.

### 3.2. Prepartum Behavior

**Figure 2:**
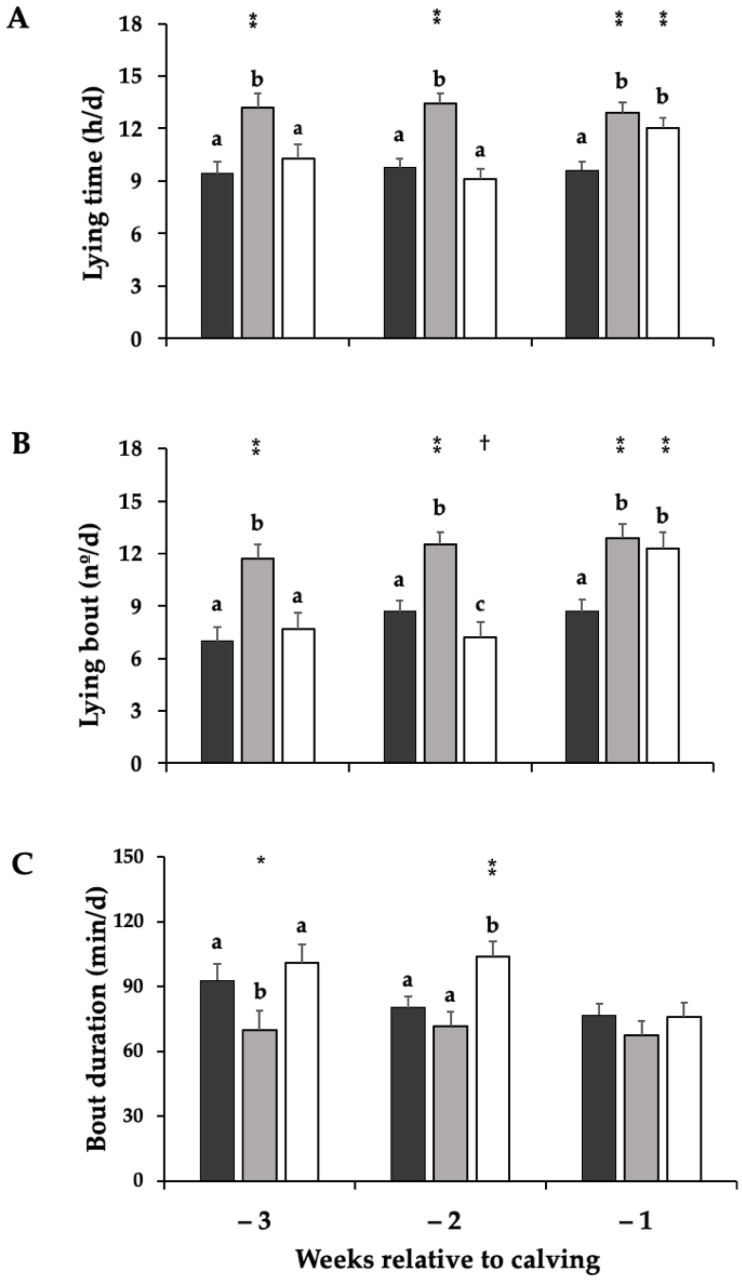
Lying time (**A**), lying bouts (**B**) and lying bout duration (**C**) of dairy cows kept in an outdoor paddock from 3 weeks before calving until calving day (black bar), or subjected to an early (3 weeks before calving; gray bar) or late movement (1 week before calving; white bar) to an indoor maternity pen where they remained until calving day (*n*: 13 cows per treatment). Least squares means and SE are reported. Different letters (a, b, c) indicate a statistical difference in the same week. † *p <* 0.10, * *p* < 0.05, ** *p* < 0.001.

The lying time differed among treatments (Figure 2A). During week −3 and −2 before calving, OP and LM cows spent approximately 3 h/d lesser time lying than EM cows (week −3 = 9.4 ± 0.7 h/d, 10.3 ± 0.8, and 13.2 ± 0.8; week −2 = 9.8 ± 0.5 h/d, 9.1 ± 0.6, and 13.3 ± 0.6, respectively; *p* < 0.001). During the week before calving, cows in the OP treatment showed a decreased lying time versus LM (9.6 ± 0.5 vs. 11.9 ± 0.6 h/d; *p* < 0.001), but there was no difference between EM and LM cows (12.9 ± 0.6 vs 11.9 ± 0.6 h/d; *p* > 0.1).

Cows in OP and LM had lesser lying bouts compared with EM during week −3 and −2 before calving (week −3 = 7.0 ± 0.8, 7.7 ± 0.9, and 11.7 ± 0.8; week −2 = 8.7 ± 0.6, 7.2 ± 0.9, and 12.5 ± 0.7 bouts/d, respectively; *p* < 0.001; Figure 2B). We observed that for OP cows the number of lying bouts decreased in week −1 compared to LM (8.7 ± 0.7 vs. 12.3 ± 0.9 bouts/d; *p* < 0.001); this difference was no longer evident with EM cows (12.3 ± 0.9 vs. 12.9 ± 0.8 bouts/d; *p* > 0.1).

Mean lying bout duration was longer in OP and LM cows than EM cows during week −3 and −2 (week −3 = 93.0 ± 7.4, 101.3 ± 8.5, and 69.9 ± 9.0; week −2 = 80.3 ± 5.4, 104.0 ± 7.1, and 71.8 ± 6.4 min/d, respectively; *p* < 0.05; Figure 2C). During the week before calving, we did not observed differences among treatments (OP = 76.8 ± 5.5, EM = 67.7 ± 6.6, and LM = 76.0 ± 6.7; *p* > 0.1).

**Figure 3:**
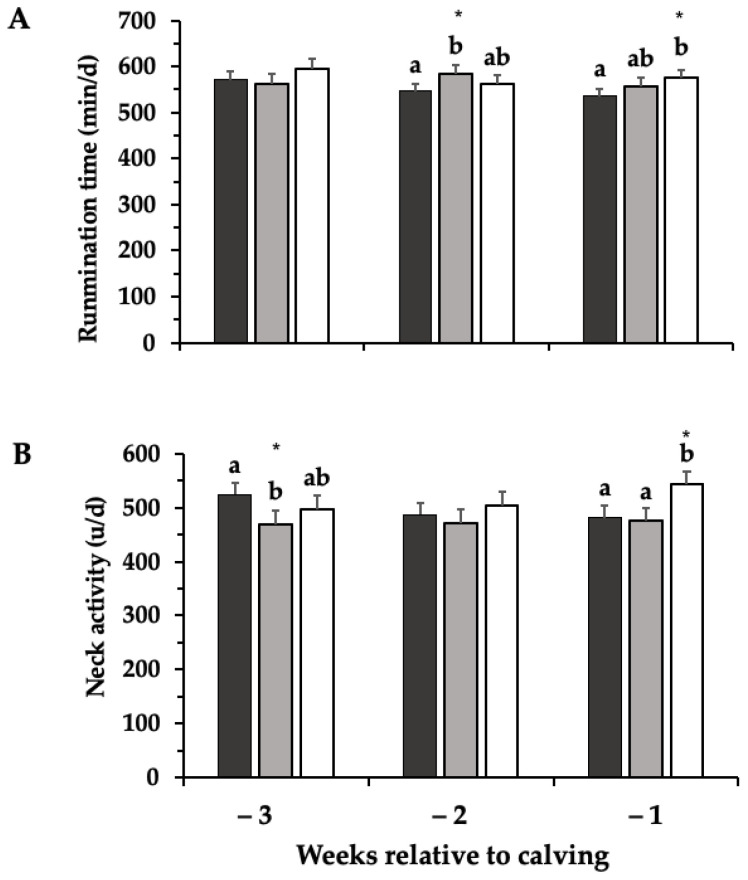
Rumination time (**A**) and neck activity (**B**) of dairy cows kept in an outdoor paddock from 3 weeks before calving until calving day (black bar), or subjected to an early (3 weeks before calving; gray bar) or late movement (1 week before calving; white bar) to an indoor maternity pen were they remained until calving day (*n*: 13 cows per treatment). Least squares means and SE are reported. Different letters (a, b) indicate a statistical difference in the same week. * *p* < 0.05.

Cows in OP had a lower daily rumination time compared to EM cows on week −2 (546 ± 16 min/d vs. 584 ± 18; *p* < 0.05) and to LM cows on week −1 (536 ± 16 min/d vs. 575 ± 19; *p* < 0.05; Figure 3A).

For daily neck activity, we found the main effects of treatment on specific weeks before calving (Figure 3B). Cows in OP had more neck activity compared to EM cows on week −3 (525 ± 22 vs. 469 ± 25 units/d; *p* < 0.05), without differences among these treatments in any other week. During week −1, LM cows showed an increase in neck activity compared to OP and EM cows (543 ± 25, 481 ± 22, and 476 ± 24 units/d, respectively; *p* < 0.05).

### 3.3. Dam and Calf Behavior

After parturition, the latency of the dam to stand after calving or to groom her calf did not differ among treatments (*p* > 0.1; Table 2). Further, there was no effect of treatment on the time that the cow spent grooming her calf (*p* > 0.1; Table 2), but self-grooming behavior was performed lesser frequently in OP dams compared to LM (*p* < 0.05; Table 3). There was no difference in self-grooming behavior between OP and EM cows (*p* > 0.1; Table 3). No differences in the number of cows that lay down after calving was found, regardless of treatment (*p* > 0.1; Table 3).

Calves from OP cows spent more time lying during the first 1.5 h after calving compared with calves from LM cows (*p* < 0.05; Table 2), but there was no difference between OP and EM treatments (*p* > 0.1; Table 2).

The number of lying bouts and latency to stand attempt was similar among treatments (Table 2; *p* > 0.1). We did not find differences in the success of calves to stand among treatments (*p* > 0.1; Table 3). However, all calves (100%) in the EM treatment and most (83%) in the LM treatment performed a suckle attempt or a successful suckling event, compared with less than a half in OP treatment (44%; *p* < 0.05; Table 3).

### 3.4. Cleanliness and Immunoglobulin G Colostrum Concentration

At enrollment, cows scored lower than three on the cleanliness score scale regardless of the treatment (*p* > 0.1; Table 4). After calving, OP cows were dirtier than EM and LM cows for all cleanliness scores (*p* < 0.05; Table 4). Furthermore, EM cows were cleaner on their upper legs than LM cows (*p* < 0.05; Table 4).

The mean IgG colostrum concentration was 22.0 ± 1.8 mg/mL (±SD; range: 1.1 to 41.4 mg/mL). The IgG colostrum concentrations were similar between OP and EM cows (25.4 ± 3.8 mg/mL vs. 20.9 ± 3.8 mg/mL; *p* > 0.1), and higher than LM cows (15.4 ± 3.8; *p* < 0.05).

## 4. Discussion

Although much research has focused on understanding the effect of the type of housing and time spent in a maternity pen in confinement-managed housed dairy cows, far less research has focused on dairy cows on pasture. In seasonal pasture dairy systems in temperate regions precalving cows might be especially affected by environmental factors such as rain, wind, or mud. For instance, in southern Chile, many farmers kept prepartum cows during late winter in outdoor paddocks with little or no opportunity to shelter, and stand-off surfaces are usually uncovered and can easily become wet and muddy if they are not well managed [7]. The objective of this study was to understand the impact of moving prepartum cows exposed to winter weather to a maternity facility at different times before calving. In addition, we investigated the effect of prepartum housing on the behavior of the dam and its offspring in the first hour after calving. As expected, we found numerous behavioral differences between cows that remained in outdoor paddocks until calving or were moved to a maternity pen in the weeks before calving. An important feature of the current study is that we also examined the effect of housing treatments on cow cleanliness levels, which was highest in the cows that calved indoors in the maternity pen. This research supports a new strategy to design calving management practices in seasonal pasture-based systems in temperate climates that minimize the effect of winter weather on cow lying behavior and calf vitality.

### 4.1. Prepartum Behavior

It is well-documented that dairy cattle spend more time lying down on dry surfaces compared with wet [37,38] or muddy ones [27]. We found that prepartum cows spent more time lying when they were housed in an indoor maternity pen (EM: 13.1 h/d and LM: 12.0 h/d) compared to when they were kept in the outdoor paddock (OP: 9.6 h/d). These results are similar to findings by Black and Krawczel (2016) [39], who observed that prepartum dairy cows spent around 10 and 13 h/d lying down when they were kept in pasture or free stall facilities, respectively. These authors [39] argued that the lower daily lying time of cows kept on pasture was due to the motivation of cows to graze. However, in our study, the cows kept in outdoor paddocks were on a surface without pasture. It is possible that the continued exposition of cows to wet, muddy and cold conditions could explain the shorter lying time. Hendriks et al. (2019) [8] observed that prepartum dairy cows managed in a pasture-based system in New Zealand spent 9.8 h/d lying down when they were exposed to winter weather. An adequate amount of time lying down is often used as an indicator of dairy cow welfare [40], and it has been described as particularly important during the latter part of pregnancy [41]. Moreover, one study found that reduced lying time caused by a lack of suitable resting areas with dry surfaces may also affect the quality of the rest [38].

In this study, we observed that access to a maternity area (both EM and LM) increased the number of transitions between standing and lying compared to the outdoor paddock (OP). Previous work reported that the dryness of the surfaces also has a marked effect on the postural changes; cows kept on wet and muddy surfaces had fewer lying bouts compared to drier ones [27,42], probably caused by discomfort in the process of lying down. Campler et al. [41] reported that soft bedding—as in our study, the sawdust bedding inside a maternity pen—improves the cow traction and facilitates her postural change from lying to standing, or vice versa.

It is worth noting that our management proposal of moving the pregnant cow from an outdoor paddock to an indoor facility the last week before calving (LM) provided an increase in lying time of 31% compared to cows that remained in the outdoor paddock (OP). One of the greatest barriers for a grazing dairy producer, in order to consider moving a precalving cow to a maternity area during the calving season, is their limited indoor accommodation and human resources [41]. An advantage of the LM management in the present study is that it can be implemented with higher practicability than the EM strategy. Further studies are needed to evaluate other management strategies (e.g., access to shelter and dry surfaces to lay down) for calving cows managed in pasture-based spring calving systems, where they are typically exposed to inclement weather.

Overall, the cows kept in outdoor paddocks (OP) ruminated ~5% less than the cows that were moved to a calving facility (EM and LM). Rumination is a health status indicator in dairy cows [43], and several studies have shown deviations in rumination time related to health disorders [44,45]. Soriani et al. [44] described, in confinement systems, that cows with reduced rumination time during the last week of pregnancy maintained a reduced rumination time after calving and presented a higher frequency of diseases. We recommend that our results should be interpreted with caution as this decrease in rumination time (~5%) may not be biologically relevant.

We also found higher neck activity in cows who were moved to maternity areas the last week before calving (LM) compared with cows that remained in their housing treatments (OP or EM); this could be a response to new housing surroundings. However, we do not know whether this behavioral change can be interpreted as something positive for the cow or not.

### 4.2. Dam and Calf Behavior

To our knowledge, this is the first study to assess the maternal behavior of dairy cows managed in a pasture-based system. We assessed the maternal behavior through latency and the amount of time that the dam spent grooming her calf. We did not find differences, among housing treatments, both in the latency of the dam to groom her calf and the time that they spent grooming it. When considering the three treatments, dams spent an average of ~56% of the time of study grooming their calves, a result similar to that described by Jensen [34] in cows managed in a confinement system, suggesting that this is a behavior of high priority for them. An early and long expression of grooming after parturition benefits both dam and calf [46]. During the grooming, the dam obtains an important analgesic effect with the ingestion of amniotic fluid [47], while that calf activity is stimulated [48]. More studies are required to understand the importance of the expression of maternal behavior in dairy cows managed in intensive production systems.

Although our study does not provide evidence to declare an association between housing treatment and lying after calving behavior, we considered it important to highlight that none of the cows kept in the maternity pen throughout the prepartum period (EM) laid down after calving. It is well known that when the dam is in a standing position during the few hours after calving, it facilitates the teat-seeking behavior of the newborn calf and, therefore, the opportune ingestion of colostrum [49]. However, the effects on the dam still are unknown.

Unfortunately, due to difficulties in continuously observing self-grooming behavior, we were unable to obtain the duration of this behavior. However, we demonstrate that self-grooming was associated with housing treatment. Only one cow that calved in the outdoor paddock (OP) expressed self-grooming, while ≥50% of cows that calved in maternity pens (EM and LM) expressed this behavior. Research in dairy cows has suggested that the expression of grooming behavior is an indicator of a positive cow affective state [50,51]. In addition, the deprivation of self-grooming after the calf is delivered has been described in dams with assisted calving, and it has been suggested to utilize this deprivation as an indicator of discomfort or pain [16]. This result highlights the relevance of providing calving facilities for the improvement of the affective state of cows when calving. We encourage more research in this area.

Calves born from cows kept in outdoor paddocks (OP) spent 80% of the time of the study lying down after calving, whereas those born from cows housed in maternity pens spent 64% (EM) and 51% (LM) of time lying down. Newborn calves exposed to cold conditions are more prone to heat loss than adult cows [52]. Likely as a thermoregulatory response, the OP calves found more protection in lying than standing posture, which is a situation of concern related to poor calf welfare. Although in our study all calves tried to stand up, only the calves born from cows moved to a maternity area at the beginning of the prepartum period (EM) achieved the behaviors “successful standing”, “suckle attempt”, and “successful suckling”. Likewise, Campler et al. [32] found that calves with an early start of standing behavior were the same as those with early suckling. The adequate ingestion of colostrum (i.e., passive transfer of immunity) is essential for calf survival. Recommendations suggest that the first ingestion of colostrum should occur within the first 4 h of life [53]. To safeguard the welfare of the calf newborn exposed to winter conditions, we decided to remove the calves from the outdoor paddock at 1.5 h after calving. The impact of early exposure to winter conditions could lead to negative effects on the calves’ health and performance. Although we did not investigate this topic, we suggest that future studies do so.

Our study has some limitations. Our results are specific to cows that were kept individually housed in indoor maternity pens or outdoor paddocks, which may not represent the conditions in commercial grazing dairy herds. In addition, due to the reduced numbers of indoor maternity pens, we used a small sample size which could have prevented us from finding differences in variables such as latency to stand up after calving, latency and duration of grooming her calf, and lying behavior in the dam and her calf. A larger sample size decreases the type II error, and, therefore, we would have a higher probability of finding differences in the variables that are more difficult to detect. Furthermore, a larger sample size would allow analyzing whether any of the housing treatments is a risk factor for the expression behavior of dam and calf. In order to evaluate the applicability of our suggestion, further studies are needed to investigate the timing to move the cow to a maternity pen in large commercial pasture-based systems.

### 4.3. Cow Cleanliness and Immunoglobulin G Colostrum Concentration

As we expected, cows moved early (EM) or late to a maternity area (LM) were cleaner after calving than those kept in the outdoor paddocks (OP). The interest in promoting good hygiene conditions aims to reduce the risk of exposure to pathogens that could cause mastitis [54] and foot diseases [55]. Additionally, cleanliness conditions have been demonstrated to be an indicator of comfort [56] and welfare in dairy cows [57]. In our study, the cows came from outdoor paddocks provided with pasture. Investigations suggest that cows with access to pasture have little dirt on their bodies [58,59]. This should explain the low level of dirtiness in the cow at enrollment of study.

Beyond the effect of housing treatment, it was a matter of concern that all the cows in our study had concentrations of IgG lower than the minimum satisfactory threshold (50 mg/mL; [60]). Therefore, none of the cows in our study provided immunologically satisfactory colostrum to the calves. In an observational study in pasture-based dairy systems, Dunn et al. [61] reported that 42% of farms produced an average of <50 mg/mL IgG; thus, we are facing a disturbing scene regarding the feeding of the newborn calf. Calves achieve adequate immunocompetence through passive transfer of immunoglobulins obtained from colostrum [62]. Immunoglobulin G is the most abundant isotype found in colostrum, and it has been determined as a marker of colostrum quality [60]. Undoubtedly, the quality of colostrum in pasture-based systems sparks many questions and challenges. We encourage further studies aimed at investigating this topic.

## 5. Conclusions

We found that cows moved to a maternity pen at the beginning of the prepartum period (EM) spent more time lying and had a higher number of lying bouts than cows kept in outdoor paddocks (OP). However—and favorably—our proposal of moving cows to a maternity pen one week before calving (LM) resulted in these cows promptly increasing their lying time and lying bouts, even resembling the cows that came from three weeks housed in maternity pens (EM). In addition, in the LM treatment, more cows performed self-grooming after calving. On the other hand, we observed that calves born from cows moved early to a maternity pen (EM) had higher vitality than those calves from cows kept in outdoor paddocks (OP). Finally, we also found that cleanliness was highest in cows that calved in the maternity pens (EM or LM). Our findings demonstrate that moving the cow, early or late, from a winter outdoor paddock to a maternity pen had positive effects on the behavioral response of the cow and her newborn calf.

## Figures and Tables

**Table 1:** Description of dam and calf recorded behaviors during the first 1.5 h after calving in all treatments.

Behavior	Classification	Definition
**Dam**		
Standing	Posture	Standing with all four limbs fully extended and perpendicular to the ground [34].
Lying down	Posture	Lying on sternal and/or lateral recumbence [34].
Grooming her calf	Maternal behavior	Standing dam’s muzzle or tongue is in physical contact with, or in close proximity of the calf’s body [34].
Self-grooming	Comfort related behavior	Dam licking herself [16].
**Calf**		
Standing	Posture	Standing with all four limbs fully extended and perpendicular to the ground [34].
Lying	Posture	Lying on sternal or lateral recumbence [34].
Lying bout	Posture	Transitions from lying to standing position [29].
Standing attempt	Vitality behavior	Calf is partially standing upright with its four limbs placed under its body, with the ventral part not touching the ground. Calf does not fully extends its limbs [32].
Successful standing	Vitality behavior	Calf is standing upright with all limbs fully extended for longer than 5 s [32].
Suckle attempt	Vitality behavior	Standing calf is positioned below standing dam with its head located under the front of the dam’s udder [32].
Successful suckling	Vitality behavior	Standing calf is positioned below standing cow with head located at the udder for more than 5 s [32].

**Table 2:** Effect of housing treatment on dam and calf behaviors during the first 1.5 h after calving (LSM ± SE).

	Housing Treatment		
Behavior	OP ^1^	EM ^2^	LM ^3^
**Dam**			
Latency to stand after calving (min)	1.4 ± 1.7 ^a^	1.0 ± 2.3 ^a^	2.6 ± 2.1 ^a^
Latency to groom her calf (min)	1.5 ± 1.7 ^a^	1.1 ± 2.3 ^a^	2.7 ± 2.1 ^a^
Duration to groom her calf (min)	47.1 ± 5.1 ^a^	53.5 ± 5.8 ^a^	50.7 ± 5.2 ^a^
**Calf**			
Lying time (min)	72.0 ± 8.8 ^a^	57.7 ± 11.8 ^a,b^	45.5 ± 10.4 ^b^
Lying bouts (No.)	2.4 ± 0.9 ^a^	3.4 ± 1.3 ^a^	3.1 ± 1.2 ^a^
Latency to stand attempt (min)	19.8 ± 4.4 ^a^	10.8 ± 6.2 ^a^	16.9 ± 5.4 ^a^

^1^ Outdoor Paddock (OP): Cows kept individually in an outdoor paddock for the last 3 weeks before their expected calving date until calving day (*n*: 9). ^2^ Early Movement (EM): Cows housed individually in an indoor maternity pen for the last 3 weeks before their expected calving date until calving day (*n*: 8). ^3^ Late Movement (LM): Cows were individually kept in an outdoor paddock during weeks −3 and −2 and moved to individual indoor maternity pens during week −1 relative to calving until the day of calving (*n*: 12). ^a,b^ Indicates a statistical difference in the same row with *p* < 0.05.

**Table 3:** Association of housing treatment on the number (and percentage) of dams and calves that performed different behaviors during the first 1.5 h after calving.

	Housing Treatment		
Behavior	OP ^1^	EM ^2^	LM ^3^
**Dam**			
Lying after calving	3/9 (33) ^a^	0/8 ^a^	4/12 (33) ^a^
Self-grooming	1/9 (11) ^a^	4/8 (50) ^a,b^	9/12 (75) ^b^
**Calf**			
Successful standing	7/9 (78) ^a^	8/8 (100) ^a^	11/12 (92) ^a^
Suckle attempt	4/9 (44) ^a^	8/8 (100) ^b^	10/12 (83) ^a,b^
Successful suckling	4/9 (44) ^a^	8/8 (100) ^b^	10/12 (83) ^a,b^

^1^ Outdoor Paddock (OP): Cows kept individually in an outdoor paddock for the last 3 weeks before their expected calving date until calving day (*n*: 9). ^2^ Early Movement (EM): Cows housed individually in an indoor maternity pen for the last 3 weeks before their expected calving date until calving day (*n*: 8). ^3^ Late Movement (LM): Cows were individually kept in an outdoor paddock during weeks −3 and −2 and moved to individual indoor maternity pens during week −1 relative to calving until the day of calving (*n*: 12). ^a,b^ Indicates a statistical difference in the same row with *p* < 0.05.

**Table 4:** Effect of housing treatment on cow cleanliness scores (LSM and SE) at enrollment of the study (3 weeks before to calving) and after calving (day 0) (*n*: 13 per treatment).

	At Enrollment			After Calving		
Cleanliness Score	OP ^1^	EM ^2^	LM ^3^	OP ^1^	EM ^2^	LM ^3^
Tail head	1.1 ± 0.2 ^a^	1.1 ± 0.2 ^a^	1.1 ± 0.2 ^a^	2.6 ± 0.6 ^b^	1.3 ± 0.2 ^a^	1.3 ± 0.2 ^a^
Upper leg	1.5 ± 0.2 ^a,c^	2.1 ± 0.3 ^a,c^	2.1 ± 0.3 ^a,c^	4.3 ± 0.2 ^b^	1.5 ± 0.3 ^c^	2.7 ± 0.3 ^a^
Ventral abdomen	1.8 ± 0.2 ^a^	1.9 ± 0.3 ^a^	2.3 ± 0.3 ^a^	4.0 ± 0.2 ^b^	1.2 ± 0.3 ^a^	1.7 ± 0.3 ^a^
Udder	1.3 ± 0.2 ^a^	1.7 ± 0.3 ^a^	1.8 ± 0.3 ^a^	3.7 ± 0.2 ^b^	1.4 ± 0.3 ^a^	1.4 ± 0.3 ^a^
Lower leg	1.9 ± 0.3 ^a^	2.5 ± 0.4 ^a^	2.6 ± 0.4 ^a^	4.8 ± 0.3 ^b^	2.5 ± 0.4 ^a^	3.0 ± 0.4 ^a^

^1^ Outdoor Paddock (OP): Cows kept individually in an outdoor paddock for the last 3 weeks before their expected calving date until calving day. ^2^ Early Movement (EM): Cows housed individually in an indoor maternity pen for the last 3 weeks before their expected calving date until calving day. ^3^ Late Movement (LM): Cows were individually kept in an outdoor paddock during weeks −3 and −2 and moved to individual indoor maternity pens during week −1 relative to calving until the day of calving. ^a,b,c^ Indicates a statistical difference in the same row with *p* < 0.05.

## Data Availability

The data can be found at https://doi.org/10.6084/m9.figshare.19119389.
